# The Efficacy of Antiseptic Treatment of Surgical Drains on Bacterial Colonisation and Surgical Site Infection Post Breast Surgery: A Systematic Review and Meta-Analysis

**DOI:** 10.7759/cureus.41585

**Published:** 2023-07-09

**Authors:** Nadia Taha, Shafiq Rahman, Andrew Kilshaw

**Affiliations:** 1 Plastic Surgery, Leeds General Infirmary, Leeds, GBR

**Keywords:** surgical drain, bacterial colonisation, surgical site infection, breast reconstruction, breast surgery

## Abstract

Surgical site infection (SSI) is a common complication in women with post-operative drains following breast surgery, with the risk being as high as 19%. The authors aimed to conduct the first meta-analysis to determine the efficacy of antiseptic treatment of drains to reduce the incidence of infections by comparing it to drains with no antiseptic coating. The Preferred Reporting Items for Systematic Reviews and Meta-Analyses (PRISMA) guidelines were followed with an extensive search of the electronic databases retrieving 114 articles. Four articles met the inclusion criteria. The primary outcome measure was the incidence of SSIs and secondary outcome measures included the incidence of bacterial colonisation in the bulb fluid and drain tube. The incidence of SSIs was significantly lower in the antiseptic group compared to the control (CI 95% 0.09 - 0.82, p = 0.02). In addition, there was a lower incidence of colonisation from both the bulb fluid and drain tube with P values of < 0.00001 and P < 0.0001 respectively. The authors report the first meta-analysis within the literature showing the efficacy of antiseptic treatment of surgical drains on colonisation and SSIs following breast surgery. More high-quality trials are recommended to further the current evidence base.

## Introduction and background

Breast cancer is the most common cancer amongst women and surgery contributes to being one of the mainstay modalities of treatment [1]. Common complications include surgical site infections (SSIs) and seroma formation. Studies have shown that the risk of SSIs following breast surgery can be as high as 19% [1]. Underlying risk factors that can exacerbate the clinical course include obesity, smoking, radiotherapy to the site, chemotherapy, and skin necrosis [2,3].

Breast reconstructive surgery can involve large tissue dissections and some surgeons to use post-operative drains. This can prevent the accumulation of fluid but increases the risk of infection [2]. The use of post-operative antibiotics until drain removal is a common practice amongst many surgeons. Antimicrobial therapy has the potential to cause adverse reactions as well as propagate antimicrobial resistance [2,4]. A meta-analysis conducted by Xue et al. looking into the risk factors contributing to SSIs, concluded that post-operative drains increase the risk [5]. Antiseptic treatment of surgical drains following mastectomies with or without prosthetic reconstruction reduces the risk of bacterial colonisation [4,6]. Studies by Stevens et al. and Hayhurst et al. demonstrated that using cranial shunt drains and ventricular drains impregnated with antibiotics or antibiotics close to the drain site reduces the rate of bacterial colonisation and SSIs [7,8].

Several studies have reported the use of antiseptic treatment of surgical drains on bacterial colonisation and surgical site infection post breast surgery [1,2,4,6]. This includes both mastectomies and reconstructive surgery. Currently, the literature is devoid of a meta-analysis to quantitatively amalgamate the results of reports comparing the use of drain antisepsis versus untreated surgical drains on the incidence of bacterial colonisation and SSIs. The authors aim to perform the first systematic meta-analysis to enhance the current evidence base on the role of drain antisepsis in mitigating the incidence of SSIs.

## Review

Methods

This systematic review and meta-analysis study was performed in line with the Preferred Reporting Items for Systematic Reviews and Meta-Analyses (PRISMA) guidelines [9]. The authors did not register the review on the Prospero database, the international prospective register of systematic reviews.

Eligibility Criteria

The eligibility criteria included all observational and randomised controlled trials of patients that underwent breast surgery with drain placement comparing drain insertion with an antiseptic coating against a comparative group with no antiseptic coating. There was no restriction on sex, age or co-morbidities. There was no exclusion on the type of antiseptic applied to the drain. Case reports, case series as well as abstracts were not included in this review. Studies not reported in English were also excluded. There was no restriction on studies that involved pre-operative antibiotic administration on induction. However, studies involving the use of post-operative antibiotics were excluded to minimise bias when assessing the efficacy of antiseptic drain coating on minimising SSIs.

Primary and Secondary Outcomes

The primary outcome measure was the incidence of SSIs and secondary outcome measures included the incidence of bacterial colonisation in the bulb fluid and drain tube.

Literature Search Strategy

The literature search was conducted independently by both NT and SR. The search included the following electronic databases: Pubmed, MEDLINE, CINAHL, and the Cochrane Central Register of Controlled Trials (CENTRAL) as well as ClinicalTrials.gov (https://clinicaltrials.gov/ct2/home). The last search was done on 14th May 2023. The search was conducted using the terms: “breast surgery”, “mastectomy”, “drain”, “antiseptic drain”, " breast reconstruction", “bacterial colonisation”, "non-antiseptic coating", “surgical site infection” and "SSI". These were combined with adjuncts of “and as well as “or”. A review of the bibliographic lists of relevant articles was also conducted.

Selection of Studies

The abstracts of the articles generated from the literature search were reviewed by authors NT and SR independently. Those which met the selection criteria were subsequently assessed for their full texts. A third author AK was consulted in cases of any discrepancy.

Data Extraction and Management

A data extraction spreadsheet was created that aligned with Cochrane's data collection form for intervention reviews. This was pilot tested in random articles and adjusted accordingly. Two authors NT and SR independently extracted and entered the data. Consultation with a third author AK was done to resolve any disagreements in the process. The data extraction spreadsheet included the study-related date, first author, year of publication, study design, the antiseptic agent used and drain care, patient's age and BMI, and primary and secondary outcome data of the studies.

Data Synthesis

Review Manager 5.4 (RevMan; The Cochrane Collaboration, 2020, Copenhagen) was used to conduct the data synthesis, and the data was entered using the random effects model. The results were reported using forest plots with 95% confidence intervals (CIs). The odds ratio (OR) was used to evaluate outcomes for dichotomous variables.

Heterogeneity Assessment

To assess the heterogeneity of studies, Cochran’s Q test (χ2) was used. In addition, the I2 assessment score was calculated to quantify any discrepancy and interpreted as follows: 0% to 25% low, 25% to 75% moderate, and 75% to 100% high heterogeneity.

Results

Literature Search Results

**Figure 1:**
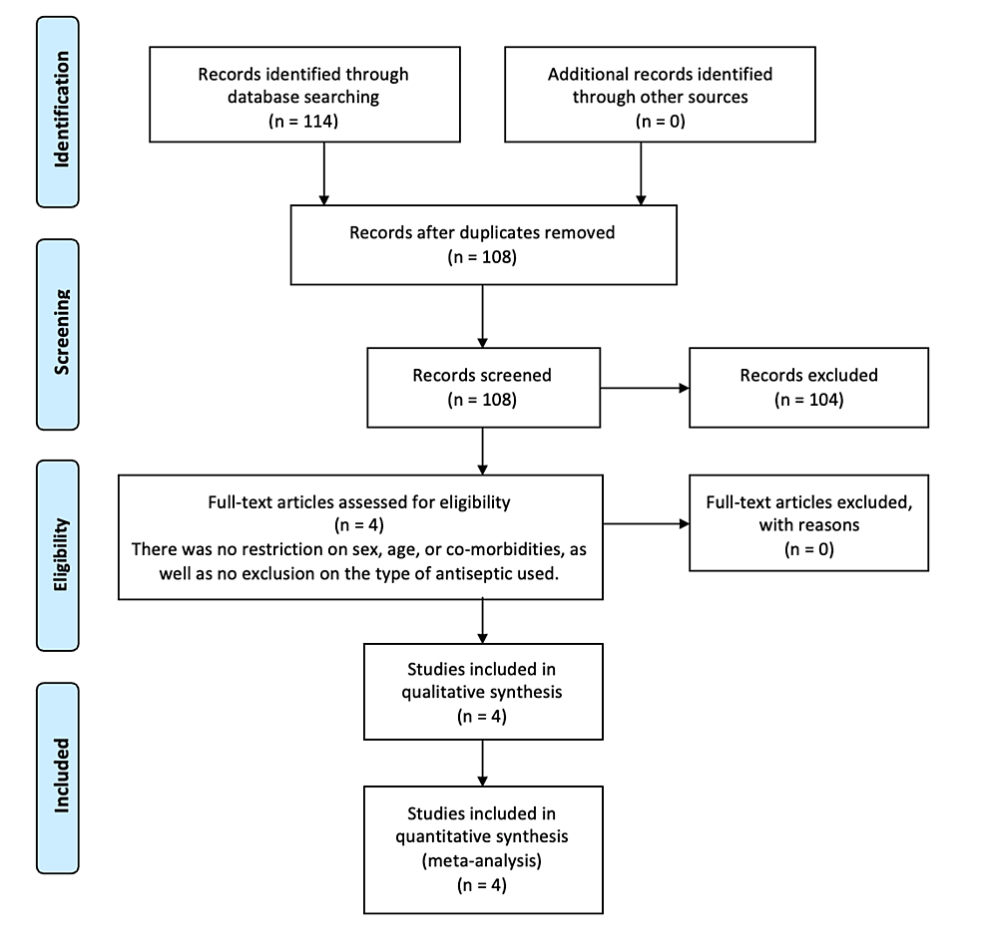
PRISMA flow diagram for this study This is the 2009 Preferred Reporting Items for Systematic Reviews and Meta-Analyses (PRISMA) flow diagram for screening and selecting articles comparing the efficacy of antiseptic treatment of surgical drains on bacterial colonisation and surgical site infection post breast surgery.

A literature search was conducted by two authors (NT and SR) independently which retrieved a total of 114 articles. Four studies were chosen based on the eligibility criteria which included three randomised control trials and one retrospective study. The search process is demonstrated in the flow chart in Figure 1.

Description of Studies

The baseline characteristics of the included studies are summarised in Table 1. With 462 drains overall, the intervention of using chlorhexidine as drain antiseptic was used in three studies and a quaternary ammonium salt (QAS-3PAC) was used in one.

**Table 1:** Demographic information and the antiseptic regime used in different studies This table is an amalgamation of demographic information across selected studies as well as group depiction for the antiseptic regime, standard drain care, and operative intervention. N/R: not reported

Author/Year	Study design	Antiseptic (agent)	Antiseptic drain care	Standard drain care	Antiseptic drain (n)/Age, years, median (range)	No antiseptic drain (n)/Age, years, median (range)	Antiseptic drain BMI mean (range)	No antiseptic drain BMI mean (range)	Operative intervention	Perioperative standardization	Surgical drain tube
Degnim et al., 2013 [4]	Randomised control trial	Chlorhexidine	1)Chlorhexidine disc at the drain exit site changed every 3 days 2)Irrigation of the drain bulb twice daily with dilute sodium hypochlorite solution	Twice daily drain cleansing with alcohol swabs	n= 67 / 61.5 (32-82)	n= 58 / 61.6 (39-84)	27.3 (15-42)	27.8 (16-45)	Total mastectomy and/or axillary lymph node dissection	Single dose of intravenous preoperative cefazolin within 30 minutes prior to skin incision.	15Fr round channel hubless drain secured with nonabsorbable monofilament suture
Degnim et al., 2014 [6]	Randomised control trial	Chlorhexidine	1)Chlorhexidine disc at the drain exit site changed every 3 days 2)Irrigation of the drain bulb twice daily with dilute sodium hypochlorite solution	Twice daily drain cleansing with alcohol swabs	n= 101 / 46 (25-67)	n= 101 / 46 (25-67)	23.8 (17-45.1)	23.8 (17-45.1)	Mastectomy and immediate reconstruction	Intravenous antibiotics within 30 minutes prior to skin incision.	N/R
Strong et al., 2018 [2]	Retrospective study	Quaternary ammonium salt, 3-trimethoxysilyl propyldimethyloctadecyl ammonium chloride (QAS-3PAC; Bio-spear)	Soaking each piece of gauze in 10mL of QAS-3PAC and left to soak for 24 hours before applying.	Soaked in non-impregnated gauze	n= 17 / 7.3 (40-79)	n= 14 / 57.7 (42-79)	26.4 (17-44)	25.4 (17-32)	Deep inferior epigastric perforator flap reconstruction or tissue expander placement	1 dose of antibiotics	N/R
Rivera-Buendía et al., 2019 [1]	Randomised control trial	Chlorhexidine	2% chlorhexidine impregnated dressing at the drain site at the end of surgery and changed 7 days post operatively.	Clean the drain exit site with 70% alcohol wipes	n= 52 / 55 +/- 13%	n= 52 52 +/- 12%	Normal 34.6% Overweight 38.5% Obesity 26.9%	Normal 28% Overweight 40.4% Obesity 30.8%	Mastectomy, breast-conserving surgery	1 dose of intravenous cephalotin 2g	N/R

1. Degnim et al. (2013) Study

Degnim et al. (2013) performed a randomised control trial including 100 patients (with 125 drains) undergoing mastectomy and/or axillary lymph node clearance. Participants were randomised to drain antisepsis in the treatment group or standard drain care in the control [4].

2. Degnim et al. (2014) Study

Degnim et al. (2014) conducted a two-sited randomised control trial that included 97 patients undergoing a bilateral mastectomy and reconstruction including 194 drains. Participants were randomised to which side (left or right) would receive the chlorhexidine-soaked drain and the contralateral side would receive standard drain care [6].

3. Strong et al. (2018) Study

Strong et al conducted a retrospective study including 20 patients undergoing either tissue expander placement or deep inferior epigastric perforator flap reconstruction with 31 surgical drains. Participants were randomised to the drains soaked in a quaternary ammonium salt, 3-trimethoxysilyl propyldimethyloctadecyl ammonium chloride (QAS-3PAC; Bio-spear) in the treatment group or non-impregnated gauze in the control group [2].

4. Rivera-Buendía et al. (2019) Study

Rivera-Buendía et al performed a randomised control trial including 104 patients undergoing breast cancer surgery with 167 surgical drains. Participants were randomised to the antiseptic dressing group with chlorhexidine or standard drain care in the control [1].

Risk of Bias Assessment

Two authors (NT and SR) independently assessed the methodological quality and risk of bias for all included articles. The Newcastle-Ottawa Quality Assessment Scale 18 was used to assess risk bias for the one observational study, demonstrated in Table 2. It involves a star scoring system where the study is judged on three domains: selection, comparability, and ascertainment of the exposure or outcome. There is a maximum of nine stars for each study. The aim is to provide a tool for the assessment of bias of the single observation study included in this review [10]. The study conducted by Strong et al. was assessed and scored well for domains of selection and outcome, but comparability was low [2]. The results included a validated measurement tool to record the incidence of bacterial colonisation with a satisfactory response rate.

**Table 2:** Newcastle-Ottawa scale quality assessment

Authors and years	Selection	Comparability	Outcome	Total
Strong et al., 2018 [2]	☆☆☆☆	☆	☆☆☆	8

The Cochrane Collaboration Tool for Risk of Bias was used in three randomised control trials demonstrated in Table 3. Overall the studies scored well. Degnim et al. (2013) demonstrated low risk in the selection and performance bias as participants were randomised by a computer programme and both groups followed the same drain care regimen [4]. The study by Degnim et al. (2014) scored a low risk for detection and attrition bias as the surgical team was blinded and all outcome data was reported [4]. Rivera-Buendía et al. demonstrated low risk for both selection and performance bias as participants were randomised using a sealed envelope, however, scored high risk for detection bias as blinding at this stage was not possible [1].

**Table 3:** The Cochrane Collaboration Tool for Risk of Bias

Author/Year	Bias	Authors judgement	Support for judgement
Degnim et al., 2013 [4]	Random sequence generation (selection bias)	Low risk	Computer randomisation programme
	Allocation concealment (selection bias)	Low risk	Computer randomisation programme
	Blinding of participants and personnel (performance bias)	Low risk	Both groups followed drain care regimens with the same instructions
	Blinding of outcome assessment (detection bias)	Unclear risk	No information given
	Incomplete outcome data (attrition bias)	Low risk	All outcome data reported
	Selective reporting (reporting bias)	Low risk	Study protocol available with no missing outcomes.
Degnim et al., 2014 [6]	Random sequence generation (selection bias)	Unclear risk	No information given on randomisation technique
	Allocation concealment (selection bias)	Unclear risk	No information given
	Blinding of participants and personnel (performance bias)	Low risk	Both groups followed drain care regimens with the same instructions
	Blinding of outcome assessment (detection bias)	Low risk	Surgical team blinded
	Incomplete outcome data (attrition bias)	Low risk	All outcome data reported
	Selective reporting (reporting bias)	Low risk	Study protocol available with no missing outcomes.
Rivera-Buendía et al., 2019 [1]	Random sequence generation (selection bias)	Low risk	Sealed envelope
	Allocation concealment (selection bias)	Low risk	Sealed envelope
	Blinding of participants and personnel (performance bias)	Low risk	Both groups followed drain care regimens with the same instructions
	Blinding of outcome assessment (detection bias)	High risk	Blinding not possible
	Incomplete outcome data (attrition bias)	Low risk	All outcome data reported
	Selective reporting (reporting bias)	Low risk	Study protocol available with no missing outcomes.

Primary Outcomes

**Figure 2:**
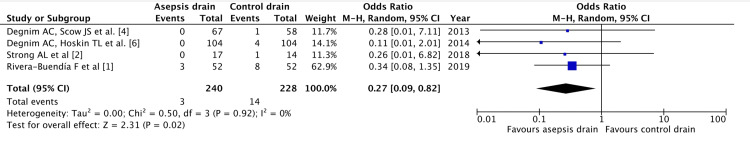
Forest plot demonstrating the incidence of surgical site infection

**Figure 3:**
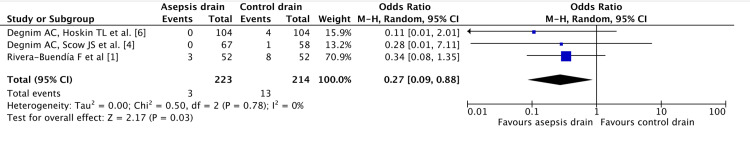
Forest plot demonstrating the incidence of surgical site infection for the studies using a chlorhexidine drain coating only

1. Surgical site infection: Incidences of surgical site infections were reported in four studies, demonstrated in Figure 2. This included 468 drains in total with 240 receiving an antiseptic coating and 228 in the control group. The random effects model was used and demonstrated a significantly lower incidence of surgical site infections within the intervention group compared to the control (CI 95% 0.09 - 0.82, P = 0.02) [1,2,4,6].

2. Sensitivity analysis: Incidences of surgical site infections in the studies using only chlorhexidine as a drain antiseptic were reported in three studies, demonstrated in Figure 3. This included 437 drains in total with 223 receiving an antiseptic coating and 214 in the control group. The random effects model was used and demonstrated a significantly lower incidence of surgical site infections within the intervention group compared to the control (CI 95% 0.09 - 0.88, P = 0.03) [1,4,6].

Secondary Outcomes

**Figure 4:**
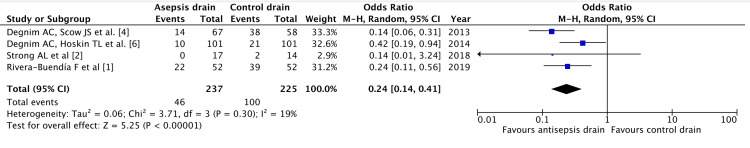
Forest plot demonstrating the incidence of colonisation in bulb fluid at 1 week

**Figure 5:**
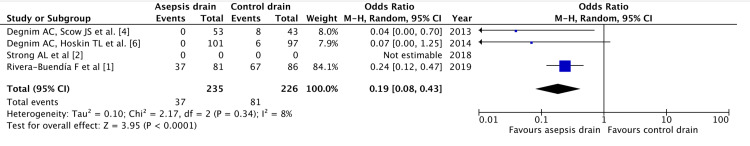
Forest plot demonstrating the incidence of colonisation from the drain tube

1. Colonisation in the bulb fluid at 1 week: Four studies reported the incidence of colonisation in the bulb fluid at one week, demonstrated in Figure 4. This included 462 drains in total with 237 receiving an antiseptic coating and 225 in the control group. The random effects model was used and demonstrated a significantly lower incidence of colonisation in drains with an antiseptic coating compared to those without (95% CI 0.14 - 0.41, P < 0.00001) [1,2,4,6].

2. Colonisation from the drain tube: Three studies reported the incidence of colonisation from the drain tube, demonstrated in Figure 5. This included 461 drains in total with 235 receiving an antiseptic coating and 226 in the control group. The random effects model was used and demonstrated a significantly lower incidence of colonisation in drains with an antiseptic coating compared to the drain group without (CI 95% 0.08 - 0.43), P < 0.0001) [1,4,6].

Discussion

The authors present the first meta-analysis within the literature assessing the efficacy of antiseptic treatment of surgical drains post breast surgery and demonstrate that local anti-septic measures result in a significant reduction in the incidence of bacterial colonisation and SSIs. On assessment, the Cochrane Q test showed no significant difference in the heterogeneity of studies, giving further consistency to the outcome variables. The Cochrane Collaboration Tool for Risk of Bias generated good overall scores, particularly in selection and performance biases in the three randomised controlled trials. The retrospective study by Strong et al. scored well on the Newcastle Ottawa scale for domains on selection and outcome, but comparability was low [2].

SSI prevention is essential in reducing healthcare costs and morbidity. The infection rates following breast reconstruction can be as high as 19% in what is considered a ‘clean surgery’ [6]. A surgical drain provides a conduit for bacterial contamination into the wound [11]. SSIs can result in implant or flap loss and patients will often require intravenous antibiotics and implant exchange or flap debridement to salvage the breast [6]. In a retrospective analysis by Reish et al. looking at the salvage rates of breast implants in patients developing an SSI following implant-based reconstructive surgery, it was found that patients admitted with a higher white cell count were at increased risk to fail implant salvage [3]. There are modifiable and non-modifiable risk factors that contribute to the risk of developing an SSI. Modifiable risk factors include diabetes and BMI, which can be optimised pre-operatively. Non-modifiable factors include the administrated of radiation and chemotherapy which are difficult to mitigate [3]. Local antiseptic coating of drains in breast surgery is simple to instigate and can assist in minimising the infection rate.

The intervention of using chlorhexidine as a drain antiseptic was used in three studies and a quaternary ammonium salt (QAS-3PAC) was used in one. Rivera-Buendía et al found that the drains were more secure with the chlorhexidine dressing applied and resulted in fewer kinks than in the standard gauze dressing [1]. Not only does the antiseptic coating to the drain reduce infection rates in the breast, but it has also been shown to reduce the risk when applied to drains in other anatomical locations [12,13]. In a randomised control trial by Timsit et al., the use of chlorhexidine-impregnated sponges on intravascular catheter dressings has been shown to reduce catheter-related infections from 1.1% to 0.5% [12]. Another randomised control trial by Levy et al. used chlorhexidine-impregnated sponges to reduce the rate of central venous catheter colonisation in infants and children after cardiac surgery, showing to reduce colonisation rates from 29% in the control group to 14.8% in the study group and reduce blood-stream infections from 5.4% to 4.2% [13].

There is widespread use of post-operative antibiotics to reduce the infection risk associated with drains following breast surgery. However, the efficacy of this is not proven and increases the burden of antibiotic resistance and side effects [4,14]. By using local drain antisepsis, the risk is avoided and is a simple intervention where side effects are uncommon and self-limiting.

This study has some limitations including a low number of randomised control trials. The use of chlorhexidine has been linked to antimicrobial resistance, but the studies involved in this study do not evaluate this. Although there is variation in the antiseptic agent used, three of the studies have used the same solution and the authors have done a sensitivity analysis on the studies using chlorhexidine drain coating alone and it has shown a significant reduction in SSIs. The authors recommend more high-quality trials assessing the efficacy of drain antisepsis in breast surgery to further evaluate its effectiveness.

## Conclusions

This meta-analysis demonstrates that the treatment of surgical drains with an antiseptic coating following breast surgery significantly reduces the incidence of bacterial colonisation and SSIs. The authors recommend further high-quality trials to improve the current evidence base.
